# Knowledge and perceptions of preconception care among health workers and women of reproductive age in Mzuzu City, Malawi: a cross-sectional study

**DOI:** 10.1186/s12978-021-01282-w

**Published:** 2021-11-14

**Authors:** Mtondera Munthali, Isabel Kazanga Chiumia, Chrispin Mandiwa, Saul Mwale

**Affiliations:** 1 Nurses and Midwives Council of Malawi, Post office Box 30361, Lilongwe, Malawi; 2 Department of Health Systems and Policy Department, College of Medicine, University of Malawi, Lilongwe, Malawi; 3 Elizabeth Glaser Pediatric AIDS Foundation, Lilongwe, Malawi; 4 Biological Sciences Department, Mzuzu University, Private Bag 201, Luwinga, Mzuzu 2, Malawi

**Keywords:** Preconception, Women of reproductive age, Health workers, Knowledge, Perception, Maternal health

## Abstract

**Background:**

Preconception care is one of the preventive strategies in maternal and new-born health as recommended by WHO. However, in sub-Saharan Africa there is poor preconception care practices. This study examined knowledge and perceptions of preconception care among health workers and women of reproductive age group in Mzuzu City, Malawi.

**Methods:**

A descriptive cross-sectional study was conducted using a mixed methods approach. Selection of respondents was done through a multistage and purposive sampling techniques respectively. A total of 253 women of reproductive age from nine townships of Mzuzu City responded to the questionnaire and 20 health workers were interviewed.

**Results:**

A total of 136 (54%) respondents had heard of preconception care. About 57.7% (n = 146) demonstrated a good level of knowledge of preconception care while 42.3% (n = 107) had poor knowledge. About 72% (n = 105) of those with good of knowledge of preconception care, lacked awareness on possibilities of talking to a health care provider on intentions of getting pregnant. About 74.7% (n = 189) of women had a positive perception towards preconception care. Knowledge of preconception care was a good predictor of positive perception (AOR = 2.5; 95% CI 1.2–5.0), however its predictability was influenced by the academic level attained. Those with secondary (AOR = 10.2; 95% CI 3.2–26.2) and tertiary (AOR = 2.3; 95% CI 1.1–4.9) were more likely to have good knowledge of preconception care than those with primary school education level. About 95% (n = 19) of health workers lacked details about preconception care but they admitted their role in preconception care.

**Conclusion:**

Preconception care practice among health workers and women of reproductive age in Mzuzu City was low. However there was positive perception towards preconception care in both parties. There is an opportunity in existing platforms for implementation of interventions targeting identified predictors for increased knowledge and uptake of preconception care.

**Supplementary Information:**

The online version contains supplementary material available at 10.1186/s12978-021-01282-w.

## Plain Language summary

World health organization defines preconception care as the provision of biomedical, behavioral and social interventions to women and couples before the occurrence of conception. In sub-Saharan Africa, there is poor preconception care practices which has resulted into high infant and maternal mortalities. This study examined the knowledge and perceptions of preconception care among health workers and women of reproductive age in order to provide evidence based outcomes for tailored interventions and policy direction. A semi-structured questionnaire and structured interviews were used to assess the knowledge level and perceptions of preconception care among women of reproductive age and health workers respectively. Of the 253 women, 57.7% showed good level of knowledge of preconception care while 42.3% had poor knowledge. Most (72%) women with good knowledge level lacked awareness that they can talk to a health worker before getting pregnant. Most (74.7%) women showed a positive perception towards preconception care which was strongly linked to having good knowledge based on the academic level attained. Those with secondary and tertiary backgrounds were more likely to have good knowledge than those with primary school level education. The majority (95%) of health workers were unable to explain well about preconception care but they admitted their role in preconception care.

**Supplementary Information:**

The online version contains supplementary material available at 10.1186/s12978-021-01282-w.

## Background

Preconception care is one of the preventive strategies in Maternal and Newborn Health (MNH) as recommended by World Health Organization (WHO) and is considered to be feasible to both developed and developing worlds [1]. MNH still remains a global health concern. WHO states that even where strong public health programme are in place across the life-course, they do not guarantee that women enter pregnancy in good health [2]. Therefore, it is necessary that certain steps should be taken before conception or early in pregnancy to maximize positive health outcomes, hence provision of preconception care is highly recommended [1]. The emerging globalization health risk factors such as obesity and tobacco use among women is common in many countries from developed to developing countries; hence just focusing on perinatal, intrapartum and postnatal is not enough to reduce the infant and maternal mortality [2]. This is why preconception care should not be limited to developed and emerging economies. It is important to take the opportunity that more women today have access to education and information, are employed, have personal income and decision-making power, and delay pregnancy, thereby having many opportunities to inform them about the need for preconception care and a healthy reproductive life [2]. Therefore, making preconception care available can have trans-generational impact on some women; those who are aware of such opportunities and can access such services.

WHO recommended implementation of preconception care 7 years ago, unfortunately there is no global consensus on the place of preconception care as part of an overall strategy to prevent maternal and childhood mortality and morbidity. In 2011 sub-Saharan Africa report on maternal health indicated a poor preconception care practice in a good number of sub-Sahara African countries largely due to low economic status, lack of health care providers, illiteracy and poor awareness about maternal health including preconception care [3]. Likewise, in Malawi there is no policy nor guidelines to direct preconception care services and there are no indicators to monitor preconception care health services [4]. Further, literature on women and health worker’s knowledge and perceptions of preconception care as an impetus for their ability to undertake or provide health care activities is limited. Despite Malawi being reported to have very high maternal and neonatal mortality rates of 439 per 100,000 and 27 per 1,000 live births respectively [4], the level of implementation and integration of PCC in its national health systems is not clear. Consequently, 33% of the pregnancies result in prematurity while 9.9% develop congenital abnormalities [5]. The content and means of delivery of preconception care are dependent on the strength of national and local health systems, and are mostly influenced by the economic realities of different countries [6], there is a need to maximize the gains from maternal and child health to ensure the attainment of Sustainable Development Goal number 3. Additionally, the knowledge level, attitude and perceptions held by the providers of preconceptions care services in different settings influences the way they engage, discuss and deliver preconception care activities to prospective parents [7]. Understanding the available platforms, knowledge level and perceptions of key actors in preconception care practice is crucial for increased uptake of tailored interventions and proper policy direction. The study therefore examined the knowledge and perceptions of preconception care among health workers and women of reproductive age group in Mzuzu City, Malawi.

## Methodology

### Study setting

**Fig. 1:**
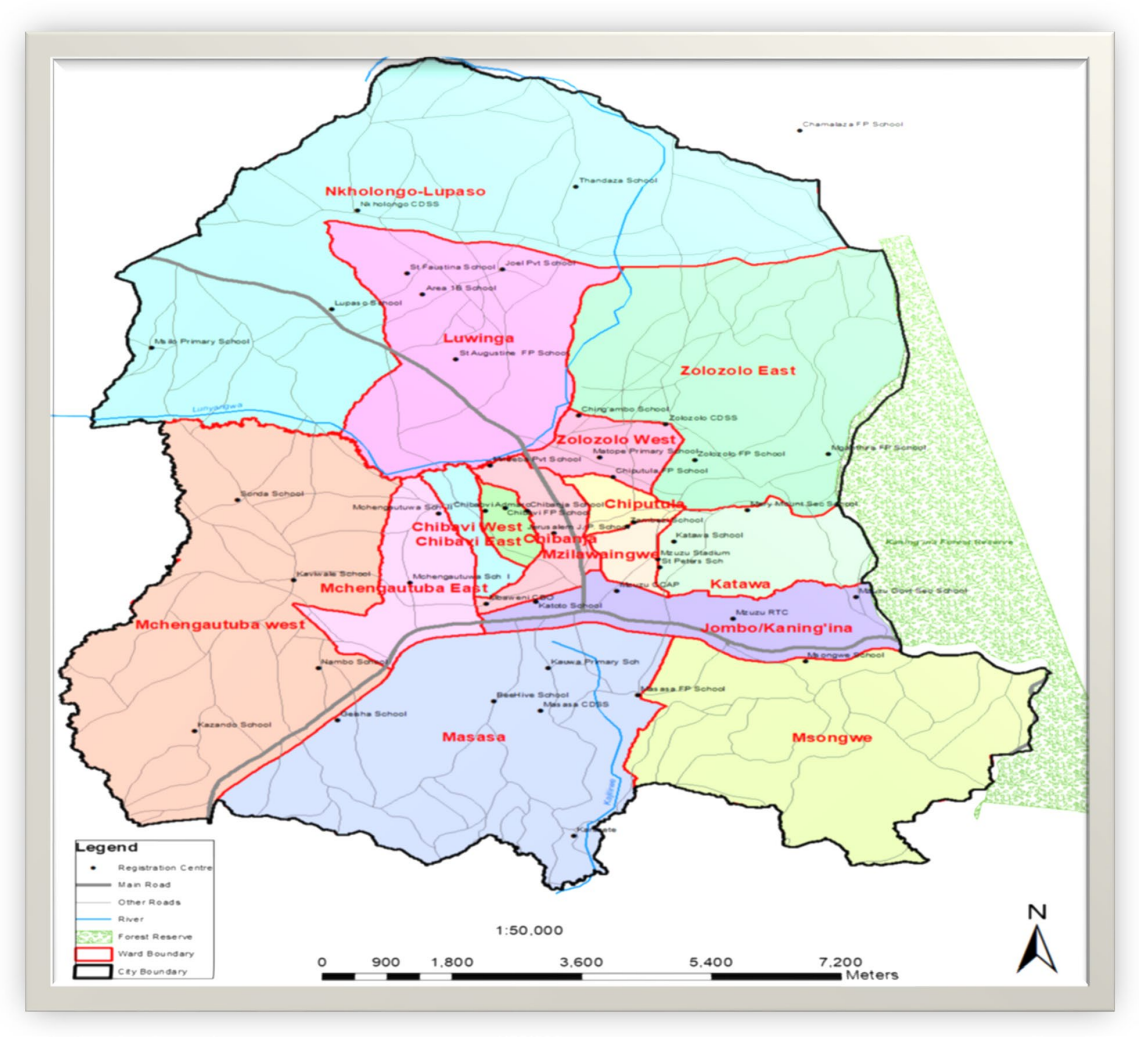
Selected study sites within Mzuzu City where respondents for the study were drawn

Malawi as a country is divided into three distinct regions namely; northern, central and southern. The northern region is reported to have the highest antenatal care coverage of 46% and 92% skilled birth attendants [8]. The study was conducted in Mzuzu City which is located in the northern region of Malawi with a land area coverage of 48 km^2^ and a population of 220,000 people [9]. Atleast 60% of Mzuzu population resides in informal settlements [9]. Study participants were drawn from the nine townships of Mzuzu City based on their geographical area population [10] namely: Chibanja, Chibavi, Mchengautuba, Katoto, Masasa, Zolozolo, Chiputula, Katawa and Luwinga (Fig. 1). The study was conducted from June 2018 to October 2019.

### Study design, sample size and sampling techniques

This was a community based cross sectional design employing a mixed method approach. Semi-structured questionnaires (see Additional file 1) and interview guides (see Additional file 2) were used as instruments for collecting quantitative and qualitative data from women of child bearing age and health workers respectively.

The minimum sample size for the study was determined by using a single population proportion formula with the following assumptions; P = 23% (50,600 women of the reproductive age group against a population of 220,000, [3], 95% level of significance (α = 0.05), Zα/2 = 1.96, 5% margin of error (d = 0.05), design effect (DEEF) of 3 and 20% non-response rate [11]. The design effect was calculated using the following formula: DEFF = 1 + **δ** (n − 1). where: **δ** = intraclass correlation coefficient, n = average size of clusters. The intraclass correlation coefficient for this study was 0.2 and the average cluster size was 6.7 giving a final DEEF of 2.6–3 [12]. The total sample size was 245. A multistage cluster sampling was employed to draw women of child bearing age from nine townships of Mzuzu City while purposive sampling technique was used to select 20 skilled birth attendants. There are basically 15 wards in Mzuzu city with at least two block leaders per ward [13]. The 15 wards were stratified based on their geographical location into three stratums of five wards each. Three wards were randomly selected from each stratum to give a total of nine wards. The selected wards were divided into clusters based on block leadership. Sample size was proportionally allocated to the selected nine wards. Selection of the respondents from the clusters were through a systematic random sampling based on a block leader’s list of women of reproductive age. Semi-structured questionnaire adapted from different literature sources [14–17] was pretested on 10% of respondents outside the target population, modified and used to collect demographic characteristics, level of knowledge on preconception care. Women’s Knowledge of preconception care was assessed using the individual respondent’s correct response to 16 items (Screening for hypertension, anaemia, diabetes mellitus, sexually transmitted infections, blood group, obesity, hepatitis B; HIV/AIDS testing and counselling; taking a balanced diet and vitamins; avoiding smoking and drinking alcohol; consulting a gynecologist or health care practitioner for advice; discussing with husband when to have a baby; having routine body exercises; awareness of issues that affect fetal development such as trauma, over the counter drugs, lack of vitamins/folic acid, natural herbs/chemicals; awareness about folic acid tablets and when they are to be taken; awareness of a baby being born with problems) [18, 19]. A score of 1 and 0 were used for correct and incorrect answers respectively. A composite knowledge score was generated through summing up 1 score for YES answers from 16 questions. Women who scored half and above (≥ 8 correct responses to the 16 questions) were regarded as ‘women with good knowledge of PCC’ whereas those who scored below 50% (< 8 incorrect responses to the 16 questions) were considered as ‘women with poor knowledge of PCC [12].

Health worker’s knowledge of preconception care was assessed using question one to six. Perception was assessed through asking women whether they felt that preconception care is beneficial; whether discussing with husband and health care worker on intentions to get pregnant is good; whether going for screening for medical conditions with husband before conception is good, whether they feel practicing family planning is good. A score of 1 and 0 was used for good and not good respectively. Women who scored 50% and above (≥ 2 out of 4 items) were rated as having positive perception and those that scored below 50% (< 2 out of 4 items) were rated as having negative perception.

### Data collection and management

The quantitative study used questionnaires [12, 20], whilst the qualitative study used interview guides for data collection. The questionnaire was developed in excel then uploaded on field task software capturing demographic data, level of knowledge on preconception care and factors that can influence access to preconception care from women of child bearing age. The questions asked were related to knowledge of folic acid, promotion of good pregnancy, factors that can affect fetal development and awareness of fetus developing congenital anomalies.

Semi-structured interviews were used to collect data on knowledge and perceptions of health workers on preconception care. Interviews were recorded using a tape recorder.

### Data analysis

Quantitative data was analysed using a statistical product for service solutions (SPSS) version 20. Descriptive statistics involved generation of frequency distributions of demographic characteristics. Inferential statistics through a Pearson Chi-square test was used to measure the association of age, marital status, education level, number of children family planning history with perception and knowledge level. A multivariable logistic regression was performed to identify factors that were significantly associated with perception and knowledge level at bivariate level of analysis (p < 0.05) to determine adjusted odds ratios (AOR). The odds ratios (OR) associated with these factors were reported as a measure of strength, together with the respective 95% confidence intervals. Qualitative data from key informant interviews transcriptions were analyzed thematically and were presented as textual expressions and direct quotations.

## Results

### Socio-demographic characteristics of respondents

**Fig. 2:**
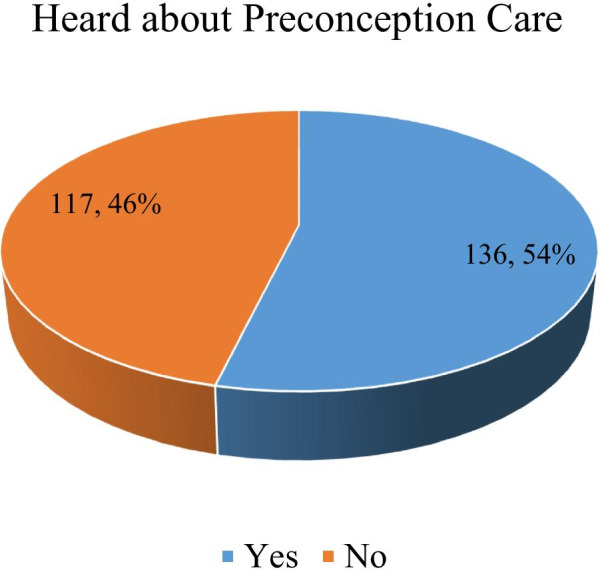
Awareness of preconception care among women of reproductive age

Among the 253 women who participated in this study, 45.8% of the women were young women aged between 15 and 24 years. At least 38.3% of women had gone up to secondary school. About 63.3% of women had children with 34% of them having 5 or more children. Nearly one third of the women (31.6%) were still in School. 48.2% of the women were married while 36% were single and 11.9% were divorced. Over half (54.2%) of the women had ever used family planning (Table 1). About 136 (54%) women of reproductive age had heard about preconception care (Fig. 2).

**Table 1:** Socio-demographic characteristics of women of reproductive age

Characteristics	Frequency	%
Age		
15–24	116	45.8
25–34	82	32.4
35–49	55	21.7
Education level		
None	5	2.0
Primary	76	30.0
Secondary	97	38.3
Tertiary	75	29.6
Occupation		
Student	80	31.6
House wife	67	26.5
Business	64	25.3
Working class	41	16.2
Marital status		
Single	91	36.0
Married	122	48.2
Divorced	30	11.9
Widowed	10	4.0
Number of children		
None	93	36.8
1 to 2	51	20.2
3 to 4	23	9.1
5 or more	86	34.0
Family planning history		
Yes	116	45.8
No	137	54.2

Out of the 20 health workers, 11 were between the ages of 20–34 representing 55%. Most health workers (11) were Nurse-Midwife Technicians (Table 2).

**Table 2:** Socio demographic data of health workers for the qualitative study

Characteristics	Frequency	%
Age		
20–34	11	55
35–40	3	15
45+	6	30
Gender		
Male	6	30
Female	14	70
Specialization		
Gynaecologist	1	5
Medical doctor	1	5
Clinical officer	1	5
Nurse-midwife officer	6	30
Nurse-midwife technician	11	55

### Predictors of PCC from bivariate and multivariable logistic regression analysis

#### Perception

About 74.7% (n = 189) of women had a positive perception towards preconception care. The association of categorical variables based on Pearson Chi-square showed that marital status (Chi-stat = 27.83, p < 0.001), occupation (Chi-stat = 9.78, p < 0.05), education level (Chi-stat = 9.62, p < 0.05) significantly associated with perception on PCC, Table 3.

**Table 3:** Predictors of PCC from bivariate and multivariable logistic regression analysis (*p < 0.05, **p < 0.01, ***p < 0.001) RC: Reference category

	Perception				Knowledge			
	Positive	Negative	Chi-stat	AOR	Good	Poor	Chi-stat	AOR
Age								
15–24^RC^	88	28			74	42		
25–34	58	24	0.88		47	35	5.67	
35–49	43	12			25	30		
Marital status								
Single^RC^	59	34	27.83***		51	40	1.63	
Married	145	15		4.29*** (CI 1.97–9.37)	104	58		
Number of children							27.43***	
None^RC^	73	20			47	46		
1 to 2	39	13			25	27		
3 to 4	18	6	1.94		7	17		
5 plus	59	25			66	18		
Occupation								
None^RC^	56	11		–	25	42		
Student	50	30	9.78*	3.27*** (CI 1.06–10.12)	67	13	37.00***	
Business	50	14		0.69 (CI 0.25–1.89)	32	32		
Employed	32	11		0.99 (CI 0.38–2.54)	21	20		
FP history								
Yes	83	34	1.37		79	38	8.95**	
No^RC^	106	30			66	70		
Education level								
None^RC^	4	1			2	2		
Primary	60	16	9.62*		25	51	45.79**	0.67 (CI 0.09–4.58)
Secondary	79	18			54	44		1.54* (CI 0.23–10.53)
Tertiary	46	29			65	10		4.72** (CI 0.58–38.09)
Perception								
Positive					96	92	11.68**	0.42*(CI 0.21–0.85)
Negative^RC^					49	16		

After multivariable logistic regression analysis was performed, marital status and occupation were found to be predictors of perception of PCC. Those that were married were more likely to have good perception towards PCC (AOR 4.2; CI 1.9–9.3) than those who were single. Those who were married and going to school were more likely to have positive perception than those who were housewives (AOR 3.2; CI 1.0–10.1).

#### Knowledge

About 57.7% (n = 146) demonstrated a good level of knowledge of preconception care while 42.3% (n = 107) had poor knowledge. There was no association between age and level of knowledge on PCC (Table 3). There was also no association between marital status and level of knowledge on PCC. Number of children is highly associated with level of knowledge on PCC (Chistat = 27.43, p < 0.001). Occupation was also highly associated with level of knowledge on PCC (Chistat = 37.00, p < 0.001). History of family planning use is associated with level of knowledge on PCC (Chistat = 8.95, p < 0.01). Education level was associated with level knowledge on PCC (Chistat = 45.79, p < 0.01). Perception was associated with level of knowledge on PCC (Chistat = 11.68, p < 0.01).

After multivariate analysis was performed perception and level of education were found to be predictors of level of knowledge of preconception care. Those that had good knowledge were more likely to have a positive perception towards PCC (AOR 0.4; CI 0.2–0.8). The higher the level of education the greater the level of knowledge. Those with tertiary education were more likely to have good knowledge than those that had none (AOR 4.7; CI 0.5–38.0).

Most women were able to outline eating a balanced diet as one of the things needed to promote a healthy pregnancy (77.0%). A few outlined engaging the hospital (29.5%), conducting exercises (27.6%), avoiding smoking and alcohol (16.3%) and taking vitamins (15.9%) as a way of promoting a healthy pregnancy. Furthermore, women outlined drinking and smoking (37.3%), over the counter drugs (22.5%), use of herbs and chemicals (20.6%), trauma (20.6%) and lack of vitamins (19.%) as some of the issues that can affect fetal development. 97.2% of the women did not know anything about folic acid and only 1.9% were able to say when it is to be taken.

Results from the qualitative interviews showed that most of the health workers knew the definition of preconception care “as the care which a woman receives before conceiving” but lacked details about the process and what services to offer to clients during preconception care (Table 4). The textual expressions of some of the health workers are reported below.

*“Well I know that preconception care is care offered to a woman before she becomes pregnant in order to correct risks that were there but as a nurse I just refer to clinicians so I do not do much (laughs*). (Respondent 3, Mzuzu Central Hospital).

*“Mmmm, preconception care is the care that is offered to women before they conceive but due to capacity it is offered to high risk women. It prepares the woman before pregnancy, prepares psychologically, prepares complications and also helps to stabilize those that have condition like hypertension before they conceive.”* (Respondent 4, Mzuzu central hospital).

**Table 4:** Health workers summarized correct responses in some key variables of preconception care

Variables	Frequency (n = 20)	%
Information to women intending to get pregnant		
Good	1	5
Poor	19	95
Management of women with chronic conditions		
Good	3	15
Poor	17	85
Management of women who delivered babies with congenital anomalies		
Good	6	30
Poor	14	70
Management of women with obstetric complications		
Good	3	15
Poor	17	85
Knowledge about preconception care		
Good	2	10
Poor	18	90
Role in preconception care	20	100
Essential	20	100
Not essential	–	–

### Roles and responsibility of health workers on preconception care

Qualitative results showed that the majority (100%) of health workers recognized that they have a role to play in preconception care services (Table 4). The respondents cited counselling, guidance and provision of the actual services, advocating with the government and other stakeholders, community sensitization and formulation of the preconception care package as some of the roles which they are supposed to do. They had a positive perception towards preconception care. Health workers felt that women also have a role to demand preconception care services and to seek the services. Some of the respondents were quoted as follows:

*“As a health worker, it’s my duty to advocate for preconception care with the government, do sensitizations of health workers, and help formulate the preconception care package.” (*Respondent 4, Mzuzu Central Hospital*).*

*“I, am responsible to give health education to women on preconception care.” Respondent 13, Mzuzu Health centre”*.

### Challenges to implementation of preconception care

We found that Health workers’ implementation of preconception care within health facilities was limited by lack of policy guidelines on PCC within the ministry of Health and in the university curricula.

*‘We never learn these things in school and to make matters worse we have no clear policy to guide us how we are to implement these services should there be.” Respondent 6, Mzuzu central.*

## Discussion

Implementation of preconception care depends on health care worker’s knowledge [19]. The study has demonstrated that the majority of health workers did not have sufficient knowledge to provide preconception care or offer care to women with complications (Table 4). This is similar to study findings in Iran [21], Canada [22], Egypt [23] and Ethiopia [12], which also reported poor knowledge level among health workers of 11.1%, 21.6%, 28.6% and 43% respectively. Further, the study has shown that the level of knowledge of preconception care was dependent on area of specialization with clinicians being knowledgeable than nurses. Similar findings on variation in knowledge and practice for preconception care among health workers were reported in Iran, Khoy city [21] where those with Bachelor of Science in family health, family physicians midwives and physicians were more knowledgeable than others. The difference in knowledge level of health workers could possibly be due to lack of standardized preconception care content incorporated in nurses and clinician training curricula. Additionally, lack of standardized guidelines on preconception care could also have contributed to this disparity in knowledge level. The capacity to offer preconception care service to clients is influenced by the health worker’s attitude, knowledge level and perceptions about PCC. This study has demonstrated that health workers had a positive perception towards preconception care as they recognized it as an essential service. This aligns well with the findings of the study that was conducted in Malawi that assessed health worker attitudes about childbearing and safer conception at two HIV clinics [24]. The study looked at health worker’s ability and willingness to counsel HIV clients on family planning methods and child spacing issues. The study revealed the willingness of health workers to discuss preconception counselling with HIV clients [24]. This is an opportunity to build on, according to the health belief model (HBM) perceived benefits can influence someone to seek or provide preconception care services.

The HBM suggests that the more knowledgeable someone is about the risk the more they are likely to engage in health promoting behaviors. Results from this study showed fair knowledge though with poor practice of seeking preconception care. Contrary to a poor knowledge level as well as poor practice of seeking preconception care reported in a study conducted in Zambia [25]. Another study in Debre Birhan town in Ethiopia also found that level of women’s knowledge of preconception care was relatively low at 17.3% [26]. Therefore, it is important to deliver health education about preconception care to women in order to increase their knowledge as well improve their health seeking behavior as many did not know it was possible to contact a health care provider before conceiving. Furthermore, studies conducted in Iran, Nepal and Sudan [27–31] showed that the women’s level of knowledge regarding preconception care is very low. The studies recommended that in settings where there is low awareness of preconception care, promotion of preconception care among reproductive age group women is important to boost maternal health care services and to reduce complications during antenatal care, institutional delivery and postnatal care [28–33]. Further, the study has established that knowledge level of preconception care among women of reproductive age group was determined by the level of education attained. The likelihood of having good knowledge level of preconception care was higher among those who attained tertiary education than those with primary education. The results are consistent with previous studies that indicated that most women with higher level of education tend to use preconception care services [14–16, 20]. Having knowledge on susceptibility of women to maternal and newborn complications can influence them to seek and demand preconception care services. Therefore, more efforts need to be taken to strengthen awareness of preconception especially to women who have primary education background as they were the ones with the least knowledge level.

## Strength of the study

The study assessed knowledge and perceptions of both women of reproductive age and health care practitioners as beneficiaries and service providers of PCC respectively for the first time in Malawi. As such the study findings can therefore serve as an impetus for enhancing the knowledge of health care practitioners in the country as well as increasing awareness of PCC among women of reproductive age.

## Limitations of the study

Since, this was a cross-sectional study that takes a snapshot of the population at a particular time limitations associated with this design are anticipated. Further, respondents could likely have suffered from some recall and social desirability bias on how to maintain a good pregnancy and issues that affect foetal development.

## Conclusion

Women of child bearing age in Mzuzu had fair preconception care knowledge though with poor or no health care seeking behavior. The level of education attained influenced their knowledge level and uptake of preconception care. Health worker’s knowledge level of preconception care was poor which affected their service provision to women needing preconception care services. Both women of reproductive age group and health workers demonstrated a positive perception towards preconception care which is an entry point to introduce such services. Therefore, there is need to provide guidelines to aid provision of preconception care services.

## Supplementary Information

**Additional file 1.** Questionnaire for women of reproductive age.

**Additional file 2.** Interview guide for health workers.

## Data Availability

The datasets used are available from the corresponding author upon reasonable request.

## Abbreviations

COMREC: College of Medicine Research Ethics Committee
FASD: Fetal alcohol spectrum disorders
GDP: Gross domestic product
HBM: Health belief model
LMIC: Low-middle income countries
MNH: Maternal and Newborn Health
MMR: Maternal mortality rate
NMR: Neonatal mortality ratio
RHD: Reproductive Health Directorate
SDG: Sustainable Development Goals
URPC: The University Research and Publication Committee
WHO: World Health Organization
