# Supplementary material for: Knowledge and perceptions of preconception care among health workers and women of reproductive age in Mzuzu City, Malawi: a cross-sectional study
Source: Reprod Health. 2021 Nov 14;18:229. doi: 10.1186/s12978-021-01282-w (PMC8591898; doi:10.1186/s12978-021-01282-w)
Supplement: Supplementary file 1 — Additional file 1. Questionnaire for women of reproductive age. [file 12978_2021_1282_MOESM1_ESM.docx]

**English version**

Assessing the Perceived Need for Preconception Care in Mzuzu City, Malawi

**Introduction**

This is an academic study that seeks to assess the perceived need for preconception care in Mzuzu City. Information obtained will be used for academic purposes only and will be treated with strict confidentiality.

Your participation in the study is voluntary. You have the right to not participate in the study or withdraw at any time you wish to do so.

Part A. **Age**

15-24 25-34 35-49

**Academic Qualification**

None

Primary School

Secondary

Tertiary

**Number of my children**

None

1-2

3-4

5+

**Occupation**

None

Business

Working class

**History of Family Planning use**

Yes No/ No

**Marriage Status**

Single

Married

Divorced

Widowed

Part B. **Please answer the following Questions**

1. What are the things that you are interested in knowing on the topic of motherhood: (conception and pregnancy?)

Nothing

Fertility issues

Congenital anomalies

Pregnancy complications

Preconception care

Other (specify)……………………………………………………………………

1. What information have you received so far?

Nothing

Fertility issues

Congenital anomalies

Pregnancy complications

Preconception care

Other (Specify) ………………………………………………………………………

1. If yes, from whom?

Health care provider

Friends

Media

Relatives

Other (specify) ……………………………………………………………………….

1. Is there something that your physician or other HCPs should have talked to you about and that you think they did not?

Yes

No

1. If yes, why do you think they do not talk to you about this issue?

Long queue

Attitude

Afraid/Shy

1. Are there questions that you wanted to ask but for some reason you didn’t?

Yes No

1. If yes, Why?

Long queue

Attitude

Afraid/ Shy

1. What can a healthy person do to promote a good pregnancy and the health of the expected baby?

Eat a balanced diet

Visit a Gynecologist/ Hospital

Take vitamins

Avoid smoking and alcohol

Exercise

Other (specify) ………………………………………………………………………..

1. What are the things that could affect the fetus’s development?

Trauma

Over the counter drugs

Natural Herbs/ Chemicals

Lack of Vitamins/ Folic acid

Drinking and smoking

Other (Specify) ………………………………………………………………………..

1. What do you know about folic acid? (When should you start taking it?)

Do not know

At least 2 months

At least 6 months

6 months +

1. Have you ever thought that the child could be born with problems?

Yes

No

1. With whom have you spoken to regarding your intention to have a baby?

No one

Husband

Family Members

Health care provider

Friends

Other (Specify) ……………………………………………………………………

1. Have you ever spoken to a health worker about intention to have a baby?

Yes

No

1. If yes, what did you ask?

Fertility Issues

Congenital anomalies

Pregnancy Complications

Preconception care

Other (Specify) ……………………………………………………………

1. If no, why you did not think to talk to him/her?

Didn’t know I can

Long queues

Afraid/ Shy

Health care Provider attitude

A taboo/ cultural Issues

Other (Specify) ……………………………………………………………

1. What do you think is the attitude of HCPs towards preconception interventions?

Negative

Positive

1. Why?

Long queues

Poor attitude

Other (Specify) ……………………………………………………………….

1. Why are so many women turning to Antenatal services only after they get pregnant?

Lack of knowledge

Taboo/cultural issues

Lack of time

Long queues at the hospital

Lack of support from husband

Other (specify) ……………………………………………………………………….

1. What was the attitude of health workers toward your questions/curiosities?

N/A

Negative

Positive

1. At this time what are the things that worry you most about motherhood (conception and pregnancy)?

Nothing

Pregnancy complications

Congenital anomalies

Other (specify) ………………………………………………………………………

1. What worries most women who intend to get pregnant?

Nothing

Pregnancy complications

Congenital anomalies

Other (specify) ………………………………………………………………………

1. Are there things/topics that you do not want to hear about? Yes No
2. If Yes what information?

Pregnancy complications

Congenital anomalies

Other (specify) ………………………………………………………………………….

1. Why?

Afraid/Shy

Taboo/cultural issues

Other (specify) …………………………………………………………………………

1. Do you have access to internet?

Yes No

1. If yes, what kind of information do you seek on the Internet?

Job search

Social media

General knowledge

Other (specify) ………………………………………………………………………

1. Have you ever sought information on the Internet on conception and pregnancy

Yes No

1. If yes, what have you searched?

Fertility issues

Congenital anomalies

Pregnancy complications

Preconception care

Other (specify) …………………………………………………………………..

1. Are there other sources of information that you have consulted on conception and pregnancy?

Yes No

1. If yes, where?

Books

Magazines

Newspaper

Friends

Health care provider

Other (specify) …………………………………………………………………

1. What kinds of information have you searched for?

Fertility issues

Congenital anomalies

Pregnancy complications

Preconception care

Other (specify) …………………………………………………………………….

**Chichewa version**

Kuunika Chiziwitso cha Kufunika Kwa Chisamaliro Kwa Amayi Asanatenge Mimba mu Mzinda wa Mzuzu, dziko la Malawi.

**Mau Otsogolera**

Uyu ndi kafukufuku wa maphudziro a ukachenjede yemwe afukuna Kuunika chiziwitso cha Kufunika kwa Chisamaliro kwa Amayi Asanatenge Mimba mu Mzinda wa Mzuzu, m’dziko la Malawi. Maganizo womwe mungapereke wokhuza kafukufukuyu azagwirizidwa ntchito pa nkhani yokhuzana ndi maphuziro basi. Ndipo maganizo onse woperekedwa uzasungidwa machinsinsi.

Kutenga nao mbali mukafukufuku si kokakamiza. Muli ndi ufulu osankha kusatenga nawo mbali.

**Gawo Loyamba**

**Nzaka zobadwa**

15-24
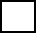
 25-34
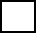
 35-49
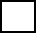


**Maphuziro anu**

Sukulu ya pulaimale
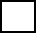


Sekondale
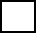


Ukachenjede
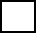


Palibe
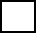


**Chiwerengero cha ana**

Ndilibe

1-2

3-4

5+

**Ntchito yomwe mumagwira**

Sindili pa ntchito

Kupanga bizinesi

Kugwira ntchito

**Mbiri yakulera**

Munayamba mwagwilitsapo njira yakulera

Eya
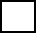
 Ayi
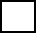


**Mbiri ya banja**

Sindinakhaleko pa banja
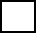


Ndili pa banja
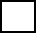


Tinasiyana
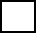


Wamasiye
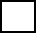


**Gawo Lachiwiri**  Chonde Yankhani Mafunso otsatirawa

1. Ndi zinthu monga ziti zomwe mungafune kuti muziwe pa nkhani yokhudza uchembele (kutenga mimba ndi kukhala ndi mimba)?

Palibe
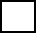


Zokhunzana ndi kubereka
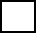


Ana kubadwa olumala
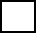


Zovuta zoza ndi mimba
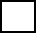


Chisamaliro kwa mayi asanatenge mimba
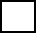


Zina (Tambasulani) ………………………………………………………………

2. Ndi uthenga wanji wokhuza uchembere womwe mwalandira padakali pano

Palibe
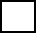


Zokhunzana ndi kubereka
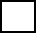


Ana kubadwa olumala
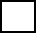


Zovuta zoza ndi mimba
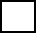


Zina (Tambasulani) ………………………………………………………………

3. Mudalandilira kuchoka kwandani?

Achipatala
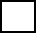


Anzanu/anthu ammudzi
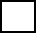


Malo owulusira ma uthenga
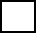


Achibale
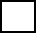


Zina (Tambasulani) ………………………………………………………………

4. Chilipo chomwe mukanakonda kuti adotolo kapena ena ogwira zachipatala?

Eya
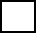


Ayi
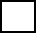


5. Mukuganiza kuti n’chifukwa chiyani sadakufotokozereni?

Nzere kutalika
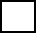


M’khalidwe
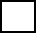


Mantha / Manyazi
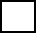


1. Alipo mafunso ena omwe mukanakonda mutafunsa koma pa zifukwa zina simunafunse?

Eya
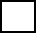
 Ayi
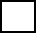


1. Ngati mwayankha eya, Chifukwa chani?

Nzere kutalika
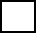


M’khalidwe la ogwira ntchito
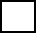


Mantha / Manyazi
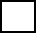


1. Ndi zinthu ziti zomwe munthu wanthazi angachite kuti apitilire kunkhara bwino ali oyembekedzera ndi kuteteza mwana oyembekelezedwa?

Kudya zamagulu asanu ndi limodzi (6)
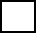


Kupita kuchipatala
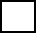


Kumwa ma vitamin
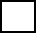


Kupewa kusuta fodya ndi kumwa mowa
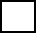


Kupanga mafizo
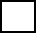


Zina (Tambasulani) ……………………………………………………………………

1. Ndi zinthu ziti zomwe zingayike pa chiopsezo mwana yemwe akuyembekezeredwa kubadwa?

Kuvulala
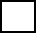


Kumwa mankhwala popanda kuunikiridwa ndi a chipatala
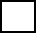


Kuperewera ma vitamin/ folic acid m’thupi
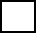


Kumwa mowa ndi kusuta fodya
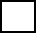


Zina (Tambasulani) ……………………………………………………………….

1. Mukuziwapo chani za mankhwala a Folic Acid? (Munthu wayenera kuyamba kumwa liti?)

Ayi sindikuziwa
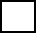


Kosachepera miyezi iwiri
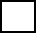


Koposera miyezi isanu ndi umodzi (6)
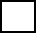


1. Munayamba mwaganiza kuti mwana atha kubadwa ndi mavuto osiyana siyana?

Eya
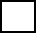


Ayi
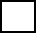


1. Munachezapo ndi ndani za malingaliro anu otenga panthupi ndi kukhala ndi mwana?

Palibe
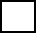


Amuna anga
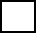


Achibale
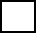


Anzanga
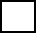


Achipatala
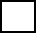


Zina (Tambasulani) ……………………………………………………………

1. Munayakhulanako ndi wazachipatala za kufuna kukhala ndi mwana?

Ayi
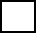


Eya
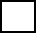


1. Mudamufunsa chiyani?

Zokhunzana ndi kubereka
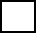


Ana kubadwa olumala
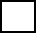


Zovuta zoza ndi mimba
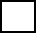


Chisamaliro kwa mayi asanatenge mimba
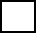


Zina (Tambasulani) ………………………………………………………………

1. Kapena ndi chifukwa chiyani simunaganineze zoyankhulana naye?

Sindimaziwa nditha kutero
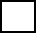


Manyazi / mantha
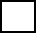


Nzere kutalika
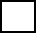


M’khalidwe laogwira ntchito
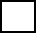


Chikhalidwe sichikundilora
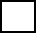


1. Chifukwa chani azimayi ambiri amapita kusikelo ya mimba pokhapokha atenga mimba osati asanatenge?

Kusaziwa
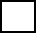


Chikhalidwe sichikundilora
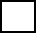


Kusowa nthawi
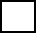


Nzere kutalika
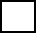


Kusowa chithandizo kuchoka kwa amuna anga
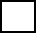


1. Padakali pano nkhawa yanu yayikulu pankhani yauchembele ndi chani?

Palibe
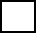


Zovuta zoza ndi mimba
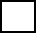


Ana obadwa ndi ulumali
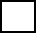


Zina (Tambasulani) ………………………………………………………………….

1. Kodi nkhawa yaikulu ya a tsikana kapena amayi ambiri omwe akufuna kutenga panthupi imakhala chani?

Palibe/ kaya
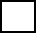


Zovuta zoza ndi mimba
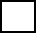


Ana obadwa ndi ulumali
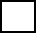


Zina (Tambasulani) ……………………………………………………………….

1. Kodi alipo mauthenga kapena nkhani zokhuza uchembere zomwe simufuna kuzimva?

Eya
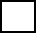


Ayi
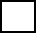


1. Ngati mwayankha eya ndi mauthenga anji?

Zovuta zoza ndi mimba
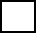


Ana obadwa ndi ulumali
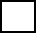


Zina (Tambasulani) …………………………………………………………

1. Chifukwa chani?

Mantha /Manyazi
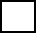


Chikhalidwe sichikundilora
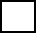


Zina (Tambasulani) ………………………………………………………….

1. Muli ndi mwayi wa internet?

Eya
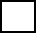


Ayi
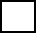


1. Ngati mwayankha eya, ndi nkhani ziti zomwe mumafufuza pa samba la internet?

Kufufuza ntchito
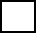


Tsamba locheza
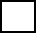


Kufufuza nzeru zosiyanasiyana
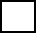


Zina (Tamabasulani) ……………………………………………………

1. Munayamba mwafufuzako nkhani za uchembele pa matsamba a internet?

Eya
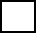
 Ayi
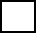


1. Ngati mwayakha eya, munafufuza chiyani?

Zokhunzana ndi kubereka
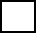


Ana kubadwa olumala
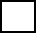


Zovuta zoza ndi mimba
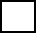


Zina (Tambasulani) ………………………………………………………………

1. Kulipo kwina komwe munafufuzako mauthenga a uchemebere?

Eya
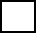


Ayi
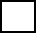


1. Ngati mwayankha eya, ndi kuti?

Mabuku
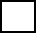


Magazine
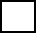


Nuzipepala
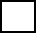


Anzanga
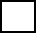


Achipatala
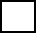


Zina (Tambasulani) ………………………………………………….

1. Ndi nkhani zanji zomwe mwafufuzapo?

Zokhunzana ndi kubereka

Ana kubadwa olumala

Zovuta zoza ndi mimba

Zina (Tambasulani) ………………………………………………………………
