# Supplementary material for: Knowledge and perceptions of preconception care among health workers and women of reproductive age in Mzuzu City, Malawi: a cross-sectional study
Source: Reprod Health. 2021 Nov 14;18:229. doi: 10.1186/s12978-021-01282-w (PMC8591898; doi:10.1186/s12978-021-01282-w)
Supplement: Supplementary file 2 — Additional file 2. Interview guide for health workers. [file 12978_2021_1282_MOESM2_ESM.docx]

**Interview Guide for Health Workers**

Date (dd/mm/yr) …../…../…..

Site ……………………………………………………………………………

Name for interviewer: ………………………………………………………..

Name of note taker: …………………………………………………………..

Start time: ……….:……. …. Finish time: ……….:……. ….

HH:MM HH:MM

INTRODUCTION

Good morning, my name is/ our Names are…………………………………………………………..

This is an academic study that seeks to assess the perceived need for preconception care in Mzuzu City. Information obtained will be used for academic purposes only and will be treated with strict confidentiality.

Your participation in the study is voluntary. You have the right to not participate in the study or withdraw at any time you wish to do so.

Part A: Demographic Data

**Age**

20-34 35-44 45-54 55-64 >64

**Professional Qualification**

Gynecology specialist Registered nurse-midwife

Doctor Nurse-midwife technician

Clinical officer

Nurse-midwife officer

1. What type of information do you give to women who come to seek family planning? (probe)
2. What type of information do you give to women who come to you with intentions of getting pregnant? (probe)
3. Let’s talk about women with chronic condition (HIV, Epilepsy, Diabetes, Hypertension etc. How you manage them? (probe)
4. Let’s talk about women how delivered babies with congenital anomalies (spinal bifida, hydrocephalus etc.) How you manage them? (probe)
5. How do you manage a woman who has experienced obstetric complications under your care? (Abortions, gestational diabetes, pregnancy induced hypertension etc.)
6. What do you know about preconception care? (probe)
7. How well can it be implemented at your facility?
8. How well can it be implemented in the communities?
9. What role do you think you have when it comes to preconception care?
10. What role do you think women of the reproductive age have on preconception care?
